# Concurrent interactome and metabolome analysis reveals role of AKT1 in central carbon metabolism

**DOI:** 10.1186/s13104-018-3364-z

**Published:** 2018-05-02

**Authors:** Nutan Gupta, Shweta Duggal, Ajay Kumar, Najmuddin Mohd Saquib, Kanury V. S. Rao

**Affiliations:** 1 Immunology Group, International Centre for Genetic Engineering and Biotechnology, Aruna Asaf Ali Marg, New Delhi, 110067 India; 2 Translational Health Science & Technology Institute, NCR, Biotech Cluster, Faridabad, 121001 India

**Keywords:** AKT1, Interactome, Metabolic flux, Central carbon metabolism, Affinity purification

## Abstract

**Objective:**

Signal transduction not only initiates entry into the cell cycle, but also reprograms the cell’s metabolism. To control abnormalities in cell proliferation, both the aspects should be taken care of, thus pleiotropic signaling molecules are considered as crucial modulators. Considering this, we investigated the role of AKT1 in central carbon metabolism. The role of AKT1 has already been established in the process of cell cycle, but its contribution to the central carbon metabolism is sparsely studied.

**Results:**

To address this, we combined the metabolomics and proteomics approaches. In accordance to our hypothesis, we found that the AKT1 kinase activity is regulating the levels of acetyl CoA through pyruvate dehydrogenase complex. Further, the decreased levels of acetyl CoA and dependency of acetyl CoA acetyl transferase protein on AKT1 kinase activity was also found to perturb the synthesis rate of palmitic acid which is a representative of fatty acid. This was analyzed in the present study using lipid labeling method through mass spectrometry.

**Electronic supplementary material:**

The online version of this article (10.1186/s13104-018-3364-z) contains supplementary material, which is available to authorized users.

## Introduction

Cell proliferation is required for the normal functioning of various tissues and perturbations in this phenomenon culminates into diseases like cancer. To proliferate, the cell requires signalling for its division as well as for generation of cell mass for the division of a mother cell into daughter cells. Both of these can be regulated at various levels by the same signaling molecule [1]. So in our quest to understand pleiotropic functions of AKT1 during proliferation, we aim to identify whether it plays any role in CCM. Thus in addition to helping the cells to progress through G1 phase of cell cycle [2], does it also help the cells to generate mass for division?

The serine-threonine kinase AKT is a key signalling molecule regulating wide range of cellular functions [3–5]. Mammalian cells express AKT as a three homologous isoforms (AKT1-3), having high degree of amino acid sequence similarity. Apoptotic rates in AKT1 deficient animal models increased thereby suggesting its role in survival [5, 6] while AKT2 exercises its control over metabolism, especially in insulin responsive tissues [5]. AKT3, along with AKT1 is implicated in mediating cell growth processes [7].

## Main text

### Methods

A stable HEK293 cell line over expressing AKT1 was generated using GATEWAY technology, as described earlier [8]. SILAC labelling was used to detect varied protein amounts among samples under different conditions. It relies on the metabolic incorporation of the given light and heavy forms of amino acids in the protein. Drug treatment experiment was performed as described earlier [8].

#### Immunoprecipitation of AKT1 and Interacting Proteins

A dual-tag affinity purification system, employing C-terminal Strep and HA tags, was used to facilitate cloning, detection, and purification of AKT1 along with its binding partners. Affinity purification using the HA tag was performed as published earlier [8]. Another aliquot of supernatant of lysed cells was used for affinity purification utilising STREP tag (Additional file 1: Method S1).

Protein Digestion and Desalting for Mass Spectrometry, Cation Exchange Protocol (P/N 4326752, AB Sciex), protein identification by Mass spectrometry and data processing in protein pilot were performed as described earlier [8].

#### Validating AKT1 interacters by western blotting

To validate AKT1 binding partners, a few proteins from AKT1 interacters list were randomly selected for reverse immunoprecipitation. The bait proteins were over expressed (as performed for AKT1), immunoprecipitated and analyzed by western blotting, using antibodies against AKT1 and relevant baits (Additional file 1: Method S1).

#### ^13^C_6_-glucose labelling and metabolite extraction

Methods employed for labelling HEK-293 cells (MK-2206 treated and untreated) with ^13^C_6_-glucose and subsequent metabolite extraction is described in Additional file 1: Method S1.

#### Metabolite Identification by Mass Spectrometry

The detailed procedure for metabolite identification through mass spectroscopy is described in Additional file 1: Method S1. The gradient profile for LC–MS/MS is provided in Additional file 3: Table S1.

#### Lipid labelling and extraction

Duplicate sets of HEK-293 cells (approximately 70% confluent) were treated overnight in the presence and absence of MK-2206. Next day a set of untreated and treated cells were trypsinized and collected as 0 h sample. For labelling lipids, another set of cells were incubated in ^13^C_6_-glucose labelled medium for 7 h, after which the cells were collected and processed for lipid extraction. Analysis for palmitic acid was performed using mass spectrometry as described in Additional file 1: Method S1.

#### Metabolite data analysis was performed as described in Additional file 1: Method S1

### Results

To understand whether AKT1 is playing any role in CCM, we first used the proteomics approach. This involved over expression of our target and its subsequent pull down so as to identify its interacting partners. In another set of samples, kinase activity of target was inhibited and was similarly processed. Then the interacting partners in both the conditions were compared to identify its kinase activity dependent interactions.

#### Identification of AKT1 binding partners

Workflow to identify binding partners of AKT1, over-expressed in HEK-293 cells, is as illustrated in Additional file 2: Figure S1. To understand kinase dependent interacters, an allosteric AKT inhibitor- MK-2206 was used [9–12]. As described in the “Methods” section, Protein lists for each treated and untreated samples for two biological samples were generated. Filters employed to generate the final list of AKT1 interacters are illustrated in Additional file 2: Figure S2. Here onwards, only those proteins which were identified in both the replicate sets were considered for further analysis. Heavy to light ratios representing differential metabolic incorporation of SILAC labels in treated and untreated samples were averaged at each merge step. A union file from all replicate data sets was generated after incorporating the mentioned filters, and the differential association values were normalized with that of AKT1.

In an effort to remove nonspecific interactors coming along with tag or affinity matrix, AKT1 was swapped with GFP in same biological system, i.e. HEK293 cells. GFP was considered since it is a non-human protein which shows minimal nonspecific binding to human proteins [13]. A total of 210 proteins were co-immunoprecipitated as eGFP interacters, in the replicate experiments (Additional file 3: Table S1). Of these, 139 proteins overlapped with AKT1 interactome list (Additional file 4: Table S2) and thus were removed as possible non-specific interacters. Finally, 904 unique interacters of AKT1 (Additional file 5: Table S3) were obtained at 95% confidence, and 1% accepted G-FDR-fit (Additional file 2: Figure S7).

**Fig. 1:**
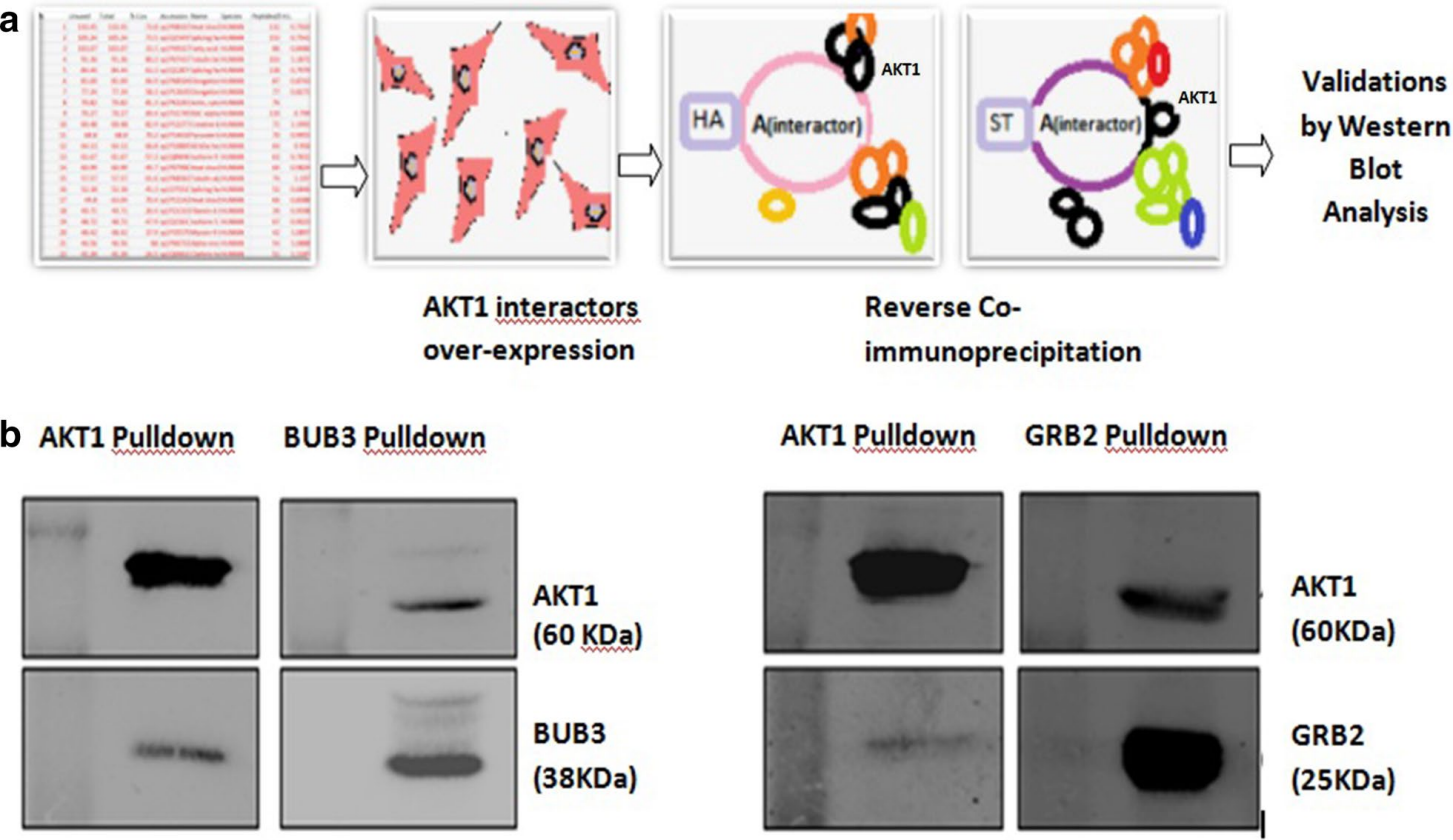
Steps employed to validate AKT1 interacters by reverse immunoprecipitation. **a** Few interacters were over-expressed in HEK293 cells and subjected to co-immunoprecipitation. **b** Western blots in panel A depict detection of BUB3 as an interacters of AKT1 in AKT1 pull down sample and parallel successful detection of AKT1 as interacters of BUB3 in BUB3 pulldown sample. Likewise, panel B depicts successful validation of GRB2 as interacters of AKT1 in AKT1 pull down sample and similarly AKT1 was found as interacters of GRB2 in GRB2 pulldown samples, respectively

As an added measure to cross examine the obtained interacters to be true AKT1 binding partners, few random interacting proteins-BUB3 and GRB2, were reverse immunoprecipitated and analyzed by western blot and these were found to be true interacting partners of AKT1 (Fig. 1).

#### Pathway enrichment of AKT1 interacters

The WebGestalt tool was used for KEGG analysis to understand the functions of AKT1 interactors [14]. Swissprot-accession IDs of the identified interactors were submitted for KEGG analysis at a stringent significance level of ≤ 10^−5^. Metabolism mainly comprising of CCM proteins was the most represented functional class. Other significantly represented functional classes included protein synthesis machinery, amino acid metabolism, signaling pathways, pathways in cancer, regulation of actin cytoskeleton, endocytosis/phagocytosis, cell cycle, etc. Details are described in Additional file 2: Figure S3.

#### Effect of AKT1 inactivation on its binding partners

Treatment with MK-2206 results in complete loss of signal for S473-specific AKT1 antibody and does not affect total AKT1 protein concentration (Additional file 2: Figure S4). This ensures that perturbations in association of interacting partners are due to the diminishing kinase activity and not due to the difference in protein concentration.

Out of 904 AKT1 interactors, 139 proteins (15.4%) showed perturbed association with AKT1-(Additional file 5: Table S3), i.e. these associations are selective for the kinase active form of AKT1. Two-fold change was considered to be significant change.

#### Kinetic flux profiling of the cell and metabolic characterization

Metabolomics approach was utilised to understand how AKT kinase activity is regulating the levels of metabolites belonging to CCM. The kinetic flux profiling was done for quantitation of cellular metabolic flux in the cultured cells. In these experiments, AKT1 over expressing cells (Untreated control cells and the MK2206 treated cells) were fed with uniformly labeled ^13^C_6_ glucose media. The 14 metabolites representing the glycolytic pathway, pentose phosphate pathway and the citrate cycle were monitored for the incorporation of ^13^C_6_ isotopomer and consumption of ^12^C isotopomer (Table 1). Specimen chromatograms are represented in Additional file 2: Figure S8. Additional file 6: Table S4 represents the relative incorporation of ^13^C isotopomer and relative consumption of ^12^C isotopomer in all four biological replicates. As the percentage of the ^13^C labelled metabolite pool increased, a concomitant reduction in the ^12^C isotopomeric population ensued. These two-parallel phenomenons kept the net concentration of metabolite pools constant as described in Additional file 2: Figure S5. Additional file 2: Figure S6 depicts possible labelling pattern of metabolites when labeled ^13^C_6_ glucose media is added to the culture.

**Table 1:** Inflow and outflow rates for the targeted metabolites in CCM pathways

Metabolite	Control (percentage incorporation/unit time)				MK-2206 treated (percentage incorporation/unit time)				Ratio (MK+/MK−)	
	^13^C	^12^C	Inflow	Outflow	^13^C	^12^C	Inflow	Outflow	Inflow	Outflow
G6P	13.333	14.286	27.619	14.286	14.286	15.385	29.670	15.385	1.074	1.077
FBP	14.286	15.789	30.075	15.789	10.526	9.615	20.142	9.615	0.670	0.609
DHAP	15.000	15.385	30.385	15.385	12.000	12.500	24.500	12.500	0.806	0.813
3PG/2PG	16.000	16.667	32.667	16.667	12.500	13.043	25.543	13.043	0.782	0.783
PEP	18.182	18.182	36.364	18.182	15.385	15.385	30.769	15.385	0.846	0.846
Pyruvate	16.364	15.385	31.748	15.385	12.000	12.500	24.500	12.500	0.772	0.813
AcoA	2.885	2.667	5.551	2.667	1.288	1.133	2.421	1.133	0.436	0.425
Citrate	2.793	5.000	7.793	5.000	1.213	4.000	5.213	4.000	0.669	0.800
OXA	0.997	0.900	1.897	0.900	0.821	0.800	1.621	0.800	0.854	0.889
R5P	6.225	6.667	12.891	6.667	4.452	6.250	10.702	6.250	0.830	0.938
IMP	0.315	0.417	0.732	0.417	0.180	0.200	0.380	0.200	0.520	0.480
AKG	0.000	0.000	0.000	0.000	0.000	0.000	0.000	0.000	1.000	1.000
Succinate	0.372	0.400	0.772	0.400	0.302	0.333	0.635	0.333	0.823	0.833
Malate	0.386	0.333	0.720	0.333	0.340	0.267	0.607	0.267	0.843	0.800

*G6P* glucose-6-phosphate, *FBP* fructose-1,6 bis-phosphate, *DHAP* dihydroxy acetone phosphate, *3PG/2PG* 3/2-phosphoglycerate, *PEP* phosphoenolpyruvate, *AcoA* acetyl-CoA, *OXA* oxaloacetate, *R5P* ribulose-5-phosphate, *IMP* inosine monophosphate, *AKG* alpha-ketoglutarate

Synthesis rate of each metabolite was calculated (experimental procedures) and correlated with association values of proteins (with AKT1) mediating the respective reactions. The levels of acetyl Co-A was found to decrease when AKT kinase activity was diminished. Acetyl CoA—a molecule connecting glycolysis and TCA has two pools in cell—the mitochondrial pool generated by PDHB using pyruvate as a substrate and cytoplasmic pool generated by citrate lyase using citrate as a substrate. In present study- metabolomics approach is not sufficient to distinguish between these two pools. Thus association of two enzymes generating them was investigated with AKT1. Association of citrate lyase was not found to be dependent on AKT1 kinase activity while the association of PDHB was found to show decreased association with AKT1. Hence these preliminary experiments suggest that AKT1 kinase activity is regulating acetyl-CoA levels through PDHB. Further we investigated the effect of decreased concentration of acetyl CoA. Association of ACAT2, (which converts acetyl-CoA to acetoacetyl-CoA) with AKT1 was found to be decreased, when AKT1 kinase activity was diminished. This may be due to availability of less substrate. Proteins showing perturbed association with AKT1 inhibition and their corresponding reaction steps are highlighted in Additional file 2: Figure S10.

#### Effect of AKT kinase activity inhibition on lipid synthesis

Decreased synthesis rate of acetyl-CoA and depleted association of PDHB and ACAT2 with AKT1 in MK-2206 treated cells suggests role of AKT1 in regulating lipid synthesis. To validate this, rate of palmitic acid synthesis (as a representative fatty acid) was determined, and was found to decrease in presence of MK2206 (Table 2). Chromatograms for this are represented in Additional file 2: Figure S9.

**Table 2:** Effect of MK2206 on the rate of fatty acid synthesis at 0 and 7 h

Metabolite	0 h		7 h			
	MK-2206(−) ^13^C (−)		MK-2206(−) ^13^C (+)		MK-2206(+) ^13^C (+)	
	Replicate1	Replicate2	Replicate1	Replicate2	Replicate1	Replicate2
^12^C (255)	4.73E+07	3.14E+07	1.22E+07	1.48E+07	5.16E+07	1.14E+08
^13^C (257)	3.10E+06	4.01E+06	8.86E+06	1.07E+07	4.80E+06	9.95E+06
^13^C (265)	4.45E+06	5.47E+06	7.93E+06	1.39E+07	1.34E+07	1.11E+07
^13^C (271)	1.31E+06	1.22E+06	4.62E+06	3.96E+06	3.49E+06	5.20E+06
% ^13^C incorporation	15.776	25.416	63.701	65.867	29.595	18.716
%^13^C (average)	20.596		64.784		24.156	

The experiment was conducted in duplicates for each condition and four separate peaks were tracked depicting ^12^C and ^13^C incorporation respectively

### Discussion

Cell proliferation manifests in the increase in cell number by an increase in the growth (i.e. increase in cell mass) and the cell division. To accomplish this cells are guided by molecular sentinels called “receptors” which receives signals from the outside world and helps the cell to decide accordingly. Cells have a smart arrangement of molecular players viz protein kinases and phosphatases, which implement the response to these signals. In our study we focused on central carbon metabolism, as it provides energy in the form of ATP and NADPH, which is crucial for every cellular activity to happen. Similar findings were also reported by other groups where metabolic phenotype in AKT2 deficient mice with prominent beta-cell dysfunction changed to type 2 diabetes because of the presence of only a single functional allele of AKT1 [15]. Such findings suggest the complexity of regulatory mechanism of AKT isoforms. In a systematic analysis of breast cancer genes for physical interactions with their interacting protein partners, PDHB was identified as a direct interacter of AKT1 [16] and similar proteins were found to interact in our study also. Our study though is not sufficient to understand whether the interaction is direct or indirect, since pulldown technique does not discriminate between these two interaction modes.

## Limitations

MK2206 is a specific allosteric inhibitor of pan AKT which do not perturb activity of any other kinase in a cell [17, 18]. Since isoform specific inhibitors are not available, we over expressed and pull down only our target to understand kinase specific perturbation in interacting partners of AKT1 and not the other isoforms. But using this approach, inhibition of basal levels of AKT2/3 may have a impact on the outcome of the results. Moreover over expression of AKT1 is changing its local concentration which may result in more interacters since interactions in cells are defined by their local concentration. But it clearly indicates that these interactions are possible in some physiological conditions.

## Additional files

**Additional file 1: Methods S1.** This document provides detailed information on the materials used and protocols followed during the experiments.

**Additional file 2: Figure S1.** Detailed workflow to identify interacting partners of AKT1 followed by their subsequent analysis. **Figure S2.** Filters employed to generate the final list of AKT1 Interactors. **Figure S3.** KEGG pathway enrichment for AKT1 binding partners using WebGestalt. **Figure S4.** Validating the effect of AKT1 inhibitor (MK-2206) on AKT1 activity by western blotting. **Figure S5.** Specimen depiction of relative incorporation of labeled carbon in G6P and FBP metabolites in MK-2206 treated and untreated samples. **Figure S6.** Possible labeling pattern for key metabolite upon feeding cells with ^13^C_6_ Glucose. **Figure S7.** Specimen chromatograms depicting estimated false discovery rates in Lys C and Trypsin digested MK-2206 treated (Lys^8^Arg^10^) and untreated (Lys^0^Arg^0^) samples. **Figure S8.** Specimen chromatograms showing metabolite peaks in the untreated and MK-2206 treated samples for five different metabolites at one of the targeted time points. **Figure S9.** Chromatograms are corresponding to ion spectra of palmitic acid (as standard) and free fatty acids. **Figure S10.** Schematic diagram of the CCM pathways depicting uptake of glucose by the cells and its subsequent utilization across different metabolites. Proteins showing perturbed association with AKT1 inhibition and their corresponding reaction steps are highlighted in red colour in figure.

**Additional file 3: Table S1.** Gradient profile for LC–MSMS method on Agilent Polaris 5NH2 2 * 150 mm.

**Additional file 4: Table S2.** Sheet1: Consolidated union file generated by integrating replicate data sets from the eGFPpull-down samples after LC–MS/MS analysis. Sheet2: Represent the common interacting partners between AKT1 and eGFP.

**Additional file 5: Table S3.** Sheet 1: Consolidated list of proteins identified as AKT1 interactors from AKT1 pull down samples after targeting HA and Strep tags. Interacting partners of AKT1 were analyzed by LC-MS/MS in duplicate and proteins identified in both replicate sets were only included to generate a union file at 1% global FDR (from fit); 95% confidence. Sheet 2: Represents the list of interacting partners showing perturbed association with AKT1 after inhibiting its kinase activity. Any protein with a heavy: light ratio of SILAC labels greater than 2and less than 0.5 was considered perturbed.

**Additional file 6: Table S4.** Consumption of ^12^C isotopomeric form in the targeted metabolites and incorporation of ^13^C in the targeted metabolites.

## Abbreviations

SILAC: stable isotope labeling with amino acids in cell culture
HA: hemagglutinin
CCM: central carbon metabolism
GFP: green fluorescent protein
FDR: false discovery rate
S473: serine 473
eGFP: enhanced green fluorescent protein
TCA: tricarboxylic acid
PDHB: pyruvate dehydrogenase
ACAT2: acetyl CoA acetyl transferase
ATP: adenosine triphosphate
NADPH: nicotinamide adenine dinucleotide phosphate
