# Supplementary material for: Concurrent interactome and metabolome analysis reveals role of AKT1 in central carbon metabolism
Source: BMC Res Notes. 2018 May 2;11:270. doi: 10.1186/s13104-018-3364-z (PMC5932847; doi:10.1186/s13104-018-3364-z)
Supplement: Supplementary file 1 — Additional file 1: Methods S1. This document provides detailed information on the materials used and protocols followed during the experiments. [file 13104_2018_3364_MOESM1_ESM.docx]

**Material and Methods:**

**Cell Culture Reagents**

Glycerol stocks of Human ORFs were obtained as pENTR221 vectors from GE Dharmacon (Human AKT1 ORF Clone ID 100067600; Human BUB3 ORF Clone ID100009502; Human GRB2 ORF Clone ID 100009383). Modified destination vector (pcDNA/FRT/TO), containing a STREP/HA Tag, was a generous gift from Dr. Matthias Mann (Max Planck Institute of Biochemistry, Germany). Flp-In TREx HEK-293 cells (#R780-07), LR Clonase II enzyme mix (#11791-100), pOG44 vector (#V6005-20), Zeocin (#46-0509), Blasticidin S (#R210-01),Hygromycin B (#10687-010) and Tetracycline (#550205) were procured from Invitrogen. Xtremegene9 (#06365787001) was from Roche. DMEM (#12800-058), RPMI 1640 (#31800-014), and RPMI [deficient in lysine and arginine] (#08-5003EA) were obtained from GIBCO. Trypsin (#CC5027.010L) and penicillin/streptomycin (#CC4007.010L) were purchased from Genetics. Normal FBS (#SH30070.03), Tetracycline (Tet) screened FBS (#SH30070.03T) and dialyzed FBS were procured from Hyclone (#SH30071.03). Glucose free RPMI 1640 (#R1383) and LysC protease (#P3428) was obtained from Sigma. D-Glucose (^13^C_6_ 99%) (#CLM-481) was procured from Euriso-Top. SILAC Labels were procured from Cambridge Isotope Laboratories, Inc. Protease inhibitor cocktail (100X) (#78429) and Pierce anti-HA agarose beads (#26182) were obtained from Thermo Scientific. MK-2206 (#S1078) was from SelleckChem; MagStrep ‘Type 2HC’ beads (#2-1612-002) were got from IBA BioTagnology. Bioconcept Labs Pvt Ltd,Gurgaon, India synthesized the HA peptide [YASFKGPNA]. Nitrocellulose membrane (#RPN303E) was purchased from GE Healthcare. Trypsin protease (#4370282) was procured from ABSciex. Full range rainbow protein molecular weight marker (#RPN800E) was from Amersham.

**Antibodies for Western Blot Analysis**

Antibodies used in the present study were: anti-HA (#SC-7392; mouse monoclonal, dilution 1:3000) from Santa Cruz; anti-AKT1 (C73H10; rabbit monoclonal, dilution 1:1000); anti-AKT (pan) (#C67E7; rabbit monoclonal, dilution 1:1000) and anti-pAKT (S473) (#D9E; rabbit monoclonal, dilution 1:1000) from Cell Signaling Technology; anti-β-Actin (#A2228; mouse monoclonal, dilution 1:15000); anti-BUB3 (#ab133699; rabbit monoclonal, dilution 1:10000) and anti-GRB2 (#ab32037; rabbit monoclonal, dilution 1:5000) from Abcam; secondary antibodies were procured from Licor Biosciences - Odyssey goat anti-mouse (#926-32210, dilution 1:15000) and Odyssey goat anti-rabbit (#926-32211, dilution 1:15000).

**Immunoprecipitation of AKT1 and Interacting Proteins**

A dual-tag affinity purification system, employing C-terminal Strep and HA tags, was used to facilitate cloning, detection, and purification of AKT1 along with its binding partners. Light and heavy labeled AKT1 expressing cells were pooled in 1:1 ratio (in equal numbers) and lysed by re-suspending in lysis buffer (150mM NaCl; 50mM Tris-HCl pH7.5; 1% NP-40; 1x protease inhibitor cocktail and 0.1M PMSF). The cell lysate was cleared by centrifugation at 13,000 rpm for 30 minutes at 4°C. Supernatant was transferred to a fresh tube and affinity purified by two different methods simultaneously. One part was kept for incubation in 200µl of pre-washed Strep-T actin magnetic beads for 1 hour at 4°C on a rotary shaker. After that, the beads were washed five times with five bead volumes of wash buffer (100mM Tris-Cl (pH 8.0), 150mM NaCl, and 1mM EDTA). Finally, the proteins were eluted twice; each elution with 150µl elution buffer [100mM Tris-Cl (pH 8.0), 150mM NaCl, 1mM EDTA, 2.5mM desthiobiotin]. The eluates were pooled and lyophilized. The other part of the cell lysate was set for incubation in 100µl of anti-HA agarose beads, pre-washed twice with TBS, for 2 hours at 4°C on an end-over-end rocker. The beads were washed thrice with five bead volumes of TBS-T buffer (TBS supplemented with 0.05% Tween-20). Proteins bound to HA beads were eluted thrice with one bead volume of 250µg/ml HA peptide per elution step. The three eluates were pooled, and lyophilized. eGFP was also over-expressed, eluted and used as an internal control to pull out any non-specific interactions.

**Protein Digestion and Desalting for Mass Spectrometry**

Samples from HA and Strep elutions were digested separately with LysC and Trypsin proteases([8](#_ENREF_8)). Each eluate obtained from treated and untreated cells resulted in a separate protein pilot output file for LysC and trypsin digestion.Untreated cells - LysC 0/0 and Trp 0/0 for HA elution and similarly for Strep elution; correspondingly LysC 8/10 and Trp 8/10 files for each HA and Strep elutions for MK-2206 treated samples were generated. Here, 0/0 and 8/10 represent SILAC labels used to differentiate untreated from treated samples respectively, while LysC and Trp represents proteases used to digest the protein samples. A union file was generated for each HA and Strep elution by merging protein pilot files obtained from LysC and Trypsin digested samples respectively. A protein was considered for analysis if it was identified in either LysC or trypsin digested samples or both. For each sample, proteins identified from HA and Strep elutions were merged to generate a single file – a protein identified in either elution was considered a hit and included for further analysis.

**Validating AKT1 Interactors by Western Blotting**

An equivalent amount of protein from immunoprecipitated samples was denatured in 1x SDS-PAGE sample buffer by heating at 95°C for 5 minutes followed by resolving them in 8-10%polyacrylamide gel in the presence of SDS. The protein bands were transferred onto nitrocellulose membrane and blocked with 1x odyssey blocking buffer (Licor Biosciences). The latter was prepared in PBS, in 1:1 ratio, by incubating overnight at room temperature (RT). Monoclonal primary antibodies were used to probe respective samples followed by compatible secondary antibodies. Odyssey direct detection imaging system from Licor Biosciences was used to perceive the results. To validate AKT1 binding partners, a few proteins from AKT1 interactor list were randomly selected for reverse immunoprecipitation. The bait proteins were overexpressed, as for AKT1, immunoprecipitated and analyzed by western blotting, using antibodies against AKT1 and the relevant baits, along with a loading control.

**^13^C_6_-glucose Labelling and Metabolite Extraction**

Methods employed for labelling HEK-293 cells with ^13^C_6_-glucose and subsequent metabolite extraction were as published earlier([13](#_ENREF_13)) . Briefly, labelled RPMI was prepared by adding ^13^C_6_-glucose to glucose free RPMI and sterilized by passing it through the0.22µm sterile filter. An hour before actual labelling, MK-2206 treated and untreated HEK-293 cells (4.5 x 10^6^) were transferred to fresh unlabeled media, to reduce perturbations in metabolite levels at the time of labelling. After that, the cells were washed once with glucose free RPMI and supplemented with fresh ^13^C_6_-glucose labelled media containing 10% dialyzed FBS, for the desired times (0, 5, 15, 30, 60, 120 minutes). The chilled mixture (1ml per sample) of methanol and water (80:20, v/v) was used to quench the metabolic reactions. After incubating the samples for 10 minutes at -20°C, the cells were scrapped and collected on ice. The cell suspension was briefly vortexed, followed by centrifugation at 6,000g for 5 minutes at 4°C, and the supernatant was stored. The pellet was re-extracted two more times at -80°C with 80% methanol. The extractions were pooled, cleared by centrifugation at 13,000g for 5 minutes and dried using speed vac. The dried samples were re-suspended in MS-grade water and centrifuged at 13,000g, 4°C for 10 minutes. Resulting supernatants were used for LC-MS/MS analysis. Each metabolite was extracted and analyzed on mass spectrometer as four separate biological replicates.

**Metabolite Identification by Mass Spectrometry**

The HPLC was coupled with a hybrid 4000 QTRAP (AB SCIEX, Foster City, CA, USA) with a Turbo V ESI ionization source interface and a computer platform equipped with a Solution Analyst software version 1.5 (ABSciex, Foster City, CA, USA) which was used for data acquisition and processing. Mass spectrometric parameters for the precursor and product ions selected in multiple reaction monitoring (MRM) for metabolites under study and their corresponding parent and daughter ions parameters were as reported earlier([13](#_ENREF_13)) . Standards, for all the metabolites under study, were purchased from Sigma Aldrich and used to generate the MRM profile and optimization of the source and compound parameters. Additional information on the validation method, the LC-gradient profile, isotopomer analysis and chromatographic approaches employed for the various metabolites were as reported earlier ([13](#_ENREF_13)).

**Lipid Labelling and Extraction**

Duplicate sets of HEK-293 cells (approximately 70% confluent) were treated overnight in the presence and absence of MK-2206. Next day a set of untreated and treated cells were trypsinizedand collected as zero hour sample. For labeling lipids, cells were incubated in ^13^C_6_-glucoselabeled medium for 7 hours, after which the labeled media was completely removed and cholate buffer (0.1M potassium phosphate, 0.05M NaCl, 5mM cholic acid, 0.1% triton) was added. The flasks were kept for incubation at RT for 5 minutes, with gentle intermittent tapping leading to cell lysis. The lysate was collected in a falcon tube and vortexed thoroughly for 5 minutes (30-second vortex followed by 30-second incubation on ice) followed by centrifugation at 3,000g for 10 minutes at 4°C. Lipids were extracted using the Bligh and Dyer protocol in borosil glass tubes([14](#_ENREF_14)). Briefly, to each 1ml of lysate collected, we added 3.75ml of 1:2 CHCl_3_:MeOH and vortexed for a minute. Subsequently, 1.25ml of CHCl_3_was added and vortexed well. This was followed by adding 1.25ml of water and vortexing. The tubes were centrifuged at 1,000rpm for 5 minutes at RT to yield a two-phase system. Lipid containing bottom layer was carefully extracted with a glass pasture pipette and dried under a stream of nitrogen gas. The dried lipid samples were re-suspended into 100µl of MS grade water, followed by adding 1ml of 4:1 ACN: 37% (v/v) hydrochloric acid. For acid hydrolysis, the sealed sample vials were kept for incubation at 90°C for 2 hours, to allow the release of fatty acids. The extracts were cooled to RT and supplemented with 1ml hexane, followed by brief vortexing for 20 seconds. The samples were not disturbed for another 5 minutes before centrifuging at 3,000g for 5 minutes. The supernatant, containing hydrolyzed lipids, was collected and another 1ml hexane was added to the pellet followed by pooling the two supernatants. The pool was dried under nitrogen stream; resuspended in a100µl mixture of chloroform, methanol, and water (50:40:5) and 0.01% aqueous ammonia; vortexed and directly used for lipid analysis.

**Analysis of Palmitic Acid**

Direct infusion method, in negative polarity, was used to analyze palmitic acid. Scan type Q1 were selected to monitor palmitic acid in the sample. The total run time of 100 cycles wasoptimized with palmitic acid [m/z=255.42 (M-H)-] standard (5µM concentration). Source parameters selected for optimization were ionisation spray voltage (IS) -4200V, CUR, GS1, GS2, 10, 20, 10, respectively (Volts). Compound dependent parameters obtained were DP, EP -20V, -10V respectively. Mass range of 250-274 Dalton (Da) with step size 0.1 Da was selected. Samples were injected into the mass spectrometer with the help of model 11 PLUS syringe pump (Harvard apparatus, Holliston, USA) at 10µl/minute flow rate. Spectra showed clear distinction between ^12^C and ^13^C-labeled palmitate.

**Metabolite Data Analysis**

From the raw data (incorporation of ^13^C and consumption of ^12^C), we first calculated the saturation percentage of the metabolites. After that, we used the slope of the graph, at half the ^13^Csaturation percentage reached by the metabolite (linear part of the graph), to obtain the net rate of incorporation for each metabolite. In the same way, we obtained the rate of consumption for a^12^Cisotope of metabolites, but unlike ^13^C-labeled metabolites, here we considered the time taken by the label to reach a minimal value.

**Details for determining the saturation concentration**

Saturation concentration of a ^13^C-labeled incorporated metabolite depicts the point of ^13^C-labeled concentration beyond which no significant change in the concentration was observed during the 120 minutes labelling period. This was identified for all metabolites by a modified *Z*-score method using the formula,

*Z*-score= (*x_i_*–*x_m_*)/ MAD,

where ‘*x_i_’* is the Concentration of ^13^C-labeled metabolites, ‘*x_m_*’ is the median of the concentration of ^13^C-labeled metabolites and ‘MAD’ is the median absolute deviation obtained by using the formula,

MAD = median{abs(*x_i_* – *x_m_*)}.

Then we calculated the rate of synthesis for individual metabolites. For this calculation, the rate of ^13^C incorporation in a given metabolite (e.g., X) gives the net rate of synthesis. That is, it represents the net outcome of both synthesis and consumption of that molecule (i.e., ^13C^-labeling rate of X = rate of synthesis – rate of consumption for the downstream step).The consumption rate of ‘X’ is directly represented by the rate of consumption of its naturally occurring isotopomer (i.e. the rate of consumption of ^12^C-labeled ‘X”). Therefore, the actual rate of synthesis of ‘X’ can be calculated as rate of ^13^C-incorporation in X + rate of decrease in concentration of the ^12^C-labeled counterpart([13](#_ENREF_13)). The actual rate of synthesis calculated for each metabolite are named here as the inflow rate (see table 1). The rate of consumption of ^12^C is recorded as the outflow rate.
