# Supplementary material for: Concurrent interactome and metabolome analysis reveals role of AKT1 in central carbon metabolism
Source: BMC Res Notes. 2018 May 2;11:270. doi: 10.1186/s13104-018-3364-z (PMC5932847; doi:10.1186/s13104-018-3364-z)

Figure S-1. Detailed workflow to identify interacting partners of AKT1 followed by their subsequent analysis; (a) HEK-293 cells over-expressing HA/Strep tagged AKT1 protein were cultured in light and heavy SILAC labeled media. MK-2206 treated and untreated cells were pooled in equal numbers and subjected to immunoprecipitation. (b) AKT1 and its interactors were affinity purified by targeting HA and Strep tags, double digested using LysC and trypsin proteases and submitted for LC-MS/MS analysis. (c) Data from LC-MS/MS analysis was analyzed using protein pilot software


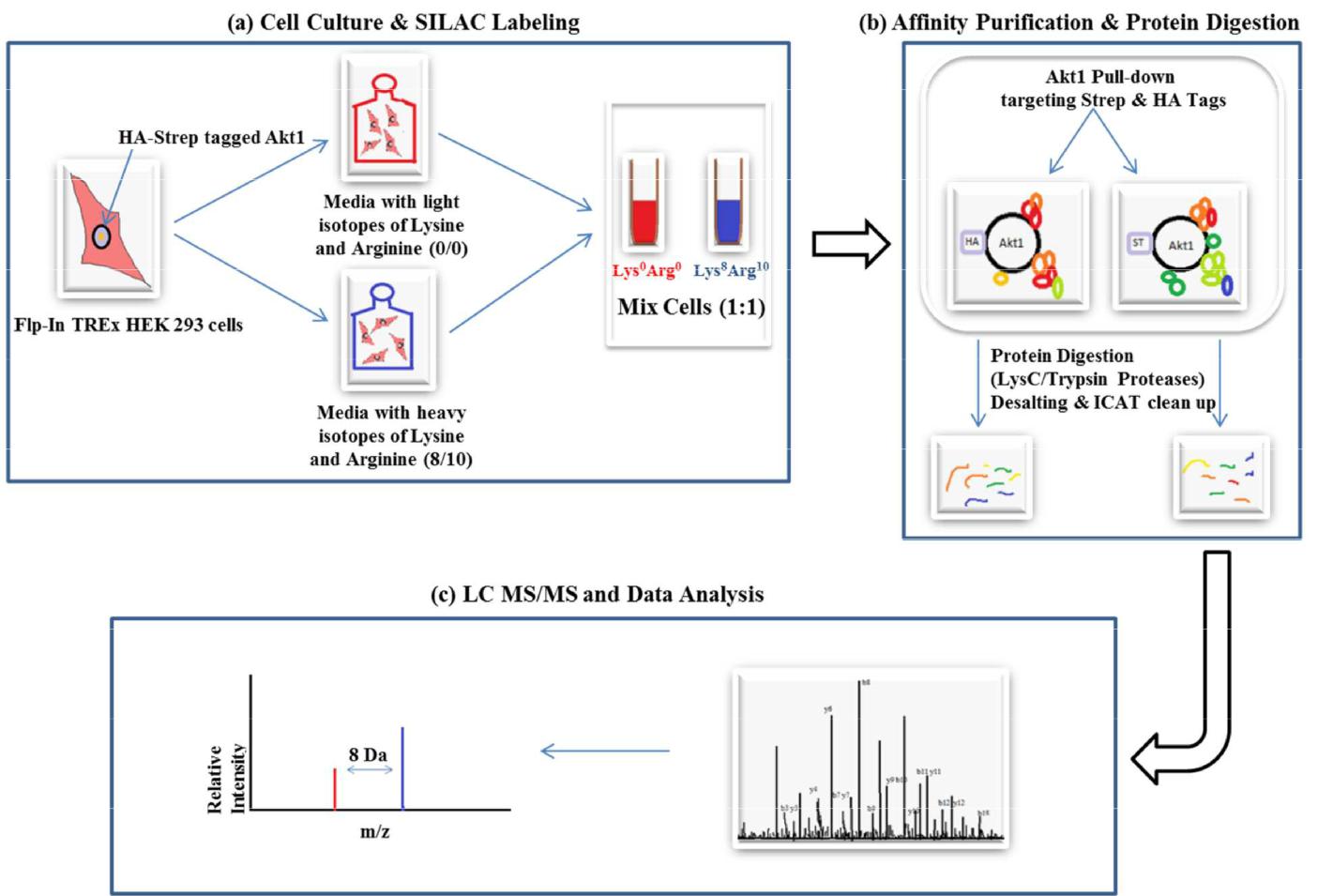


Figure S-2. Filters employed to generate the final list of AKT1 Interactors. AKT1 interactors were extracted by targeting both HA and Strep tags separately and in duplicate. Following schematic diagram depicts a methodology for either. (a) Proteins not identified in both replicate data sets for either MK-2206 untreated or treated samples were excluded from the analysis (b) Total AKT1 interactome obtained as a consequence of application of the mentioned filter was 1044 proteins (c) eGFP interactome (n= 210) was extracted in parallel and 139 proteins were identified as common interactors for both AKT1 and eGFP. These 139 proteins were substracted from AKT1 interactome, as possible non-specific interactors, and the final count of AKT1 interactors was obtained to be 905.


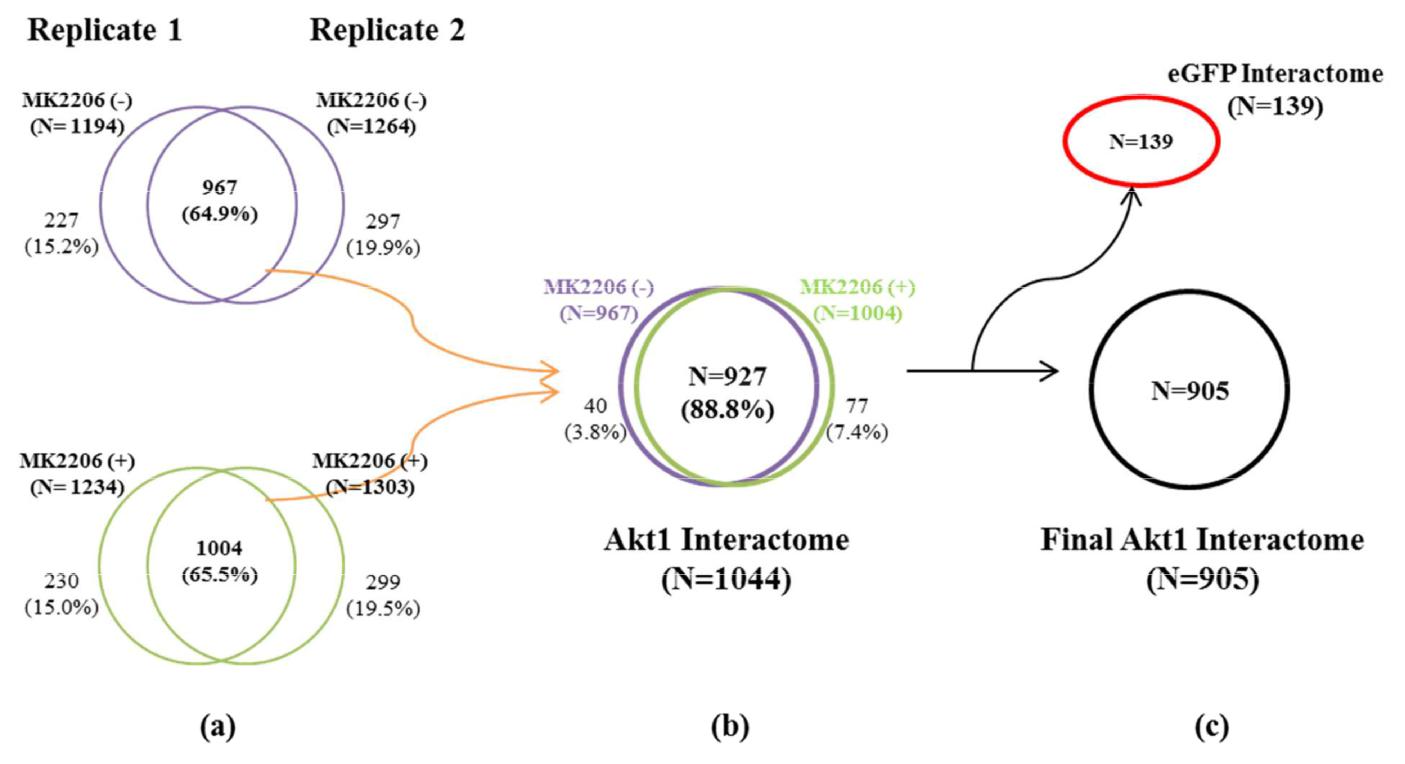


Figure S-3: KEGG pathway enrichment for AKT1 binding partners using WebGestalt. KEGG pathways enriched at a stringent p-value of 10^-5^ are only listed below. Here, KEGG pathways are aligned along the y-axis whereas x-axis represents the number of proteins representing a particular pathway.


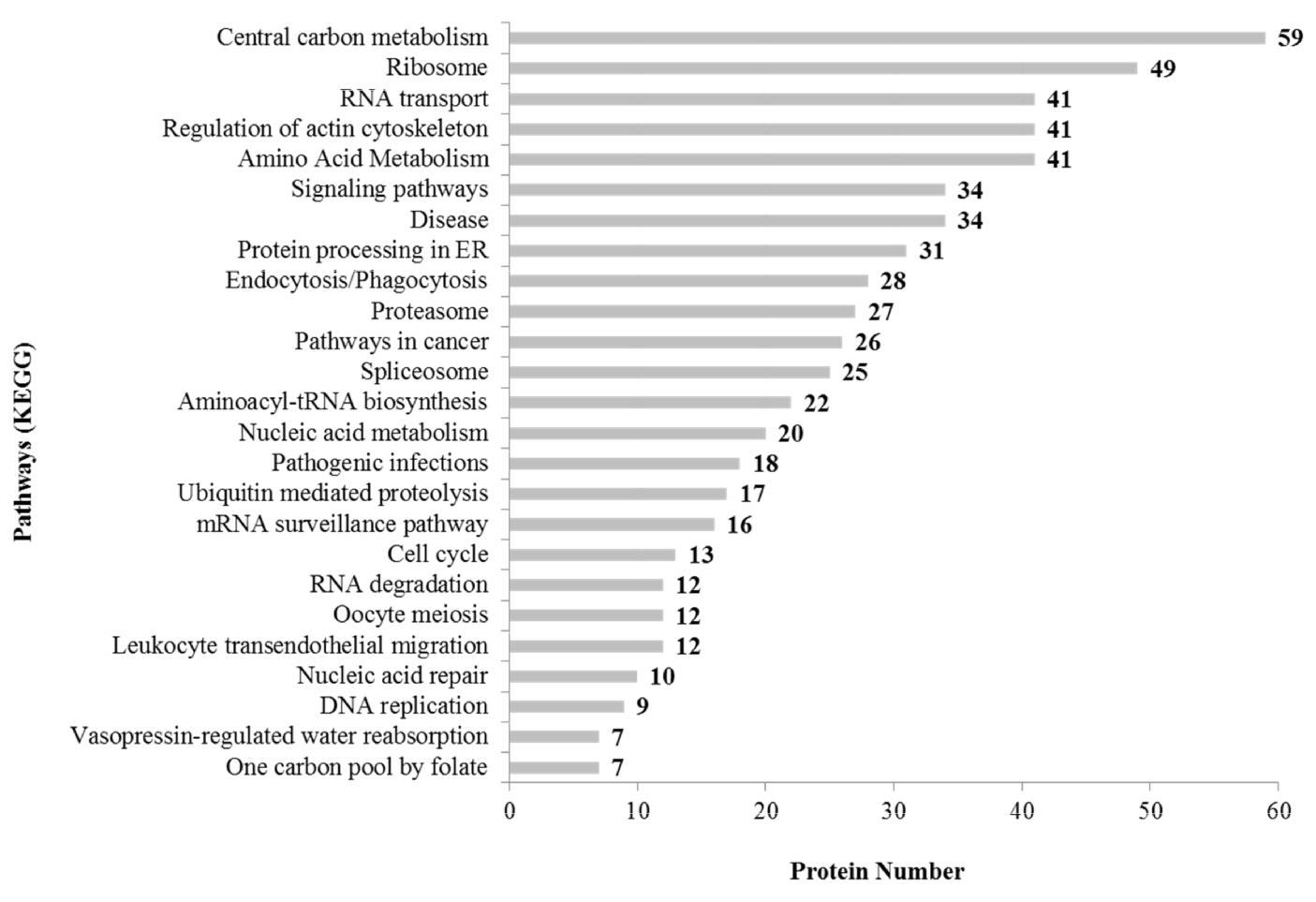


Figure S-4: Validating the effect of AKT1 inhibitor (MK-2206) on AKT1 activity by western blotting (a) MK-2206 treated samples showed no fluorescence signal when probed for AKT1 targeting one of its active sites S473. However, a positive signal was detected in untreated samples (b) when targeted for total AKT1, both MK-2206 treated and untreated samples showed positive fluorescence. Actin is used as a loading control.


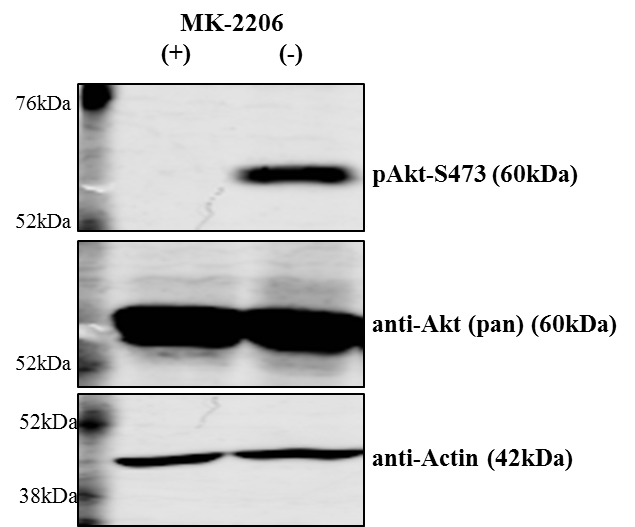


Figure S- 5: Specimen depiction of relative incorporation of labeled carbon in G6P and FBP metabolites in MK-2206 treated and untreated samples. The red curve represents the percentage incorporation of c13, and blue curve represents percentage consumption of c12.


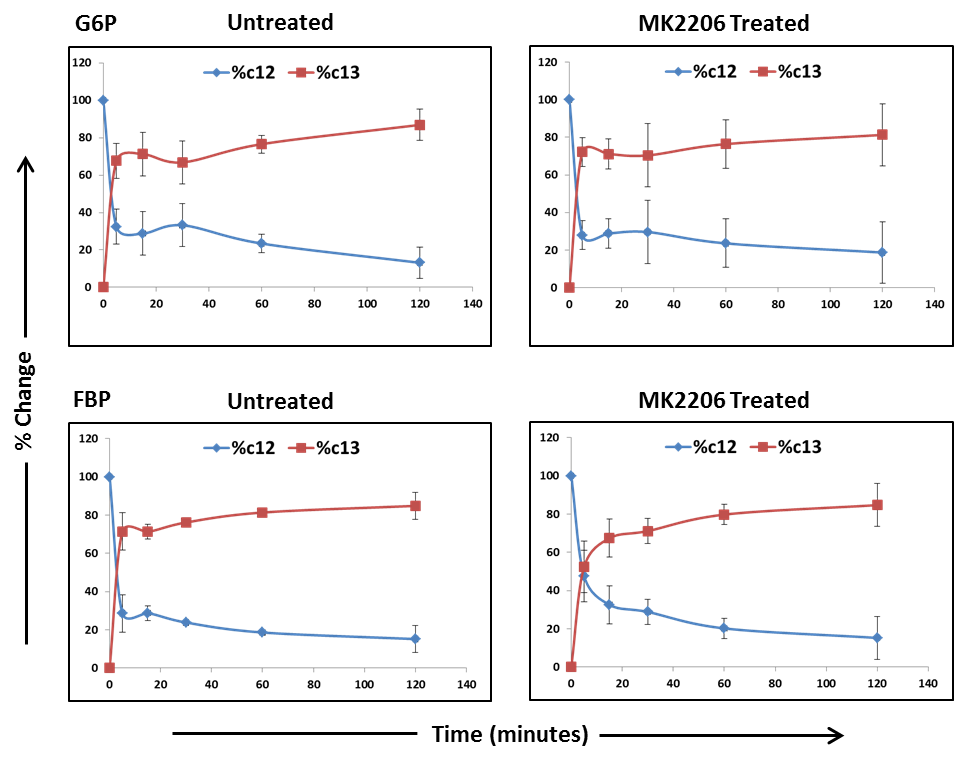


Figure S-6: Possible labeling pattern for key metabolite upon feeding cells with ^13^C_6_ Glucose. Green-C-13 and Black: natural isotope: c-12. In fumarate, the two-carbon group derived from acetate can no longer be specifically denoted; because succinate and fumarate are symmetric molecules, c-1 & c-2 are indistinguishable from c-4 & c-3.


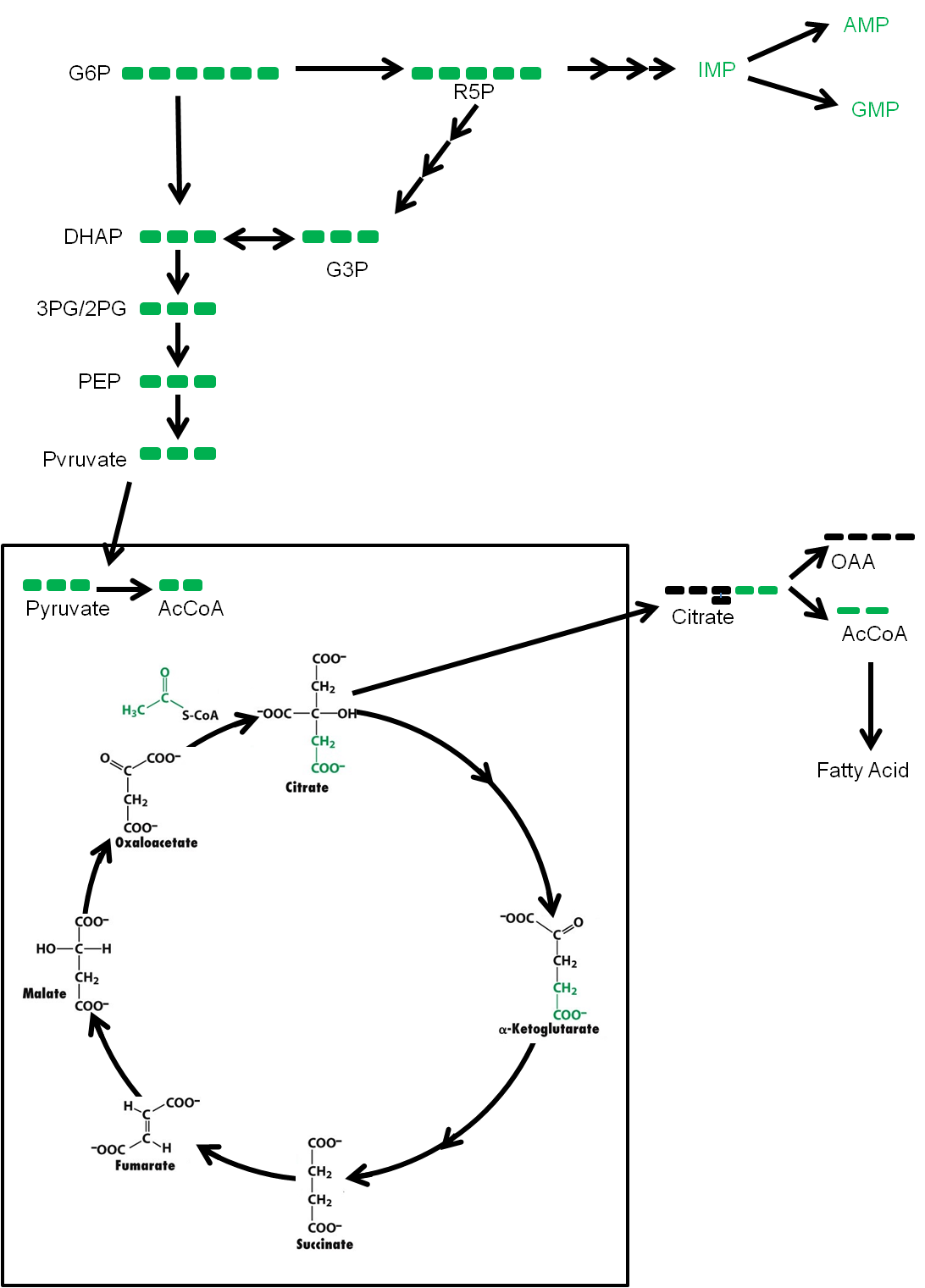


Figure S-7. Specimen chromatograms depicting estimated false discovery rates in Lys C and Trypsin digested MK-2206 treated (Lys^8^Arg^10^) and untreated (Lys^0^Arg^0^) samples


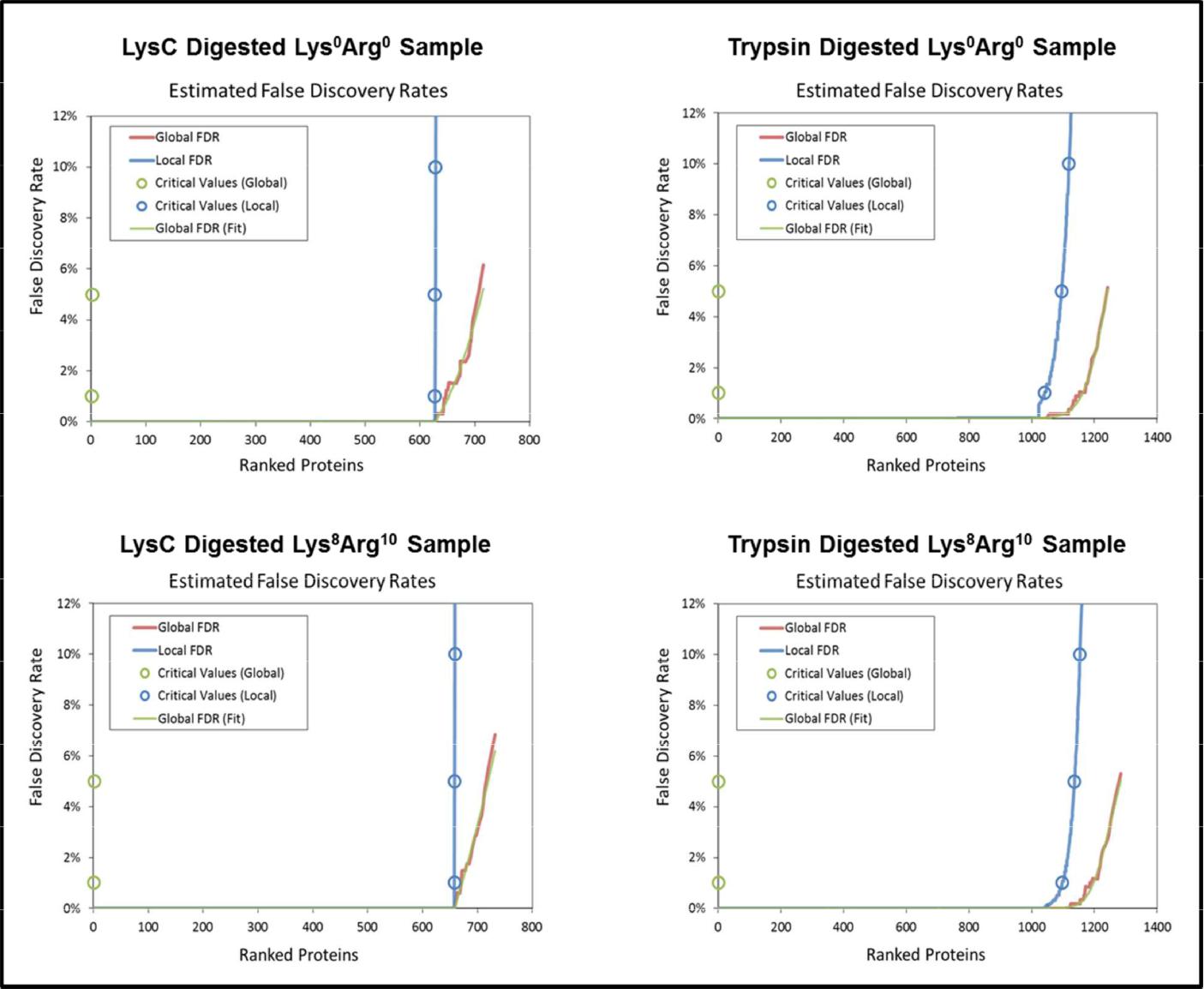


Figure S-8. Specimen chromatograms showing metabolite peaks in the untreated and MK-2206 treated samples for five different metabolites at one of the targeted time points. Time (in minutes) is represented on X-axis while Y-axis corresponds to the intensity of the identified peaks for respective metabolites.


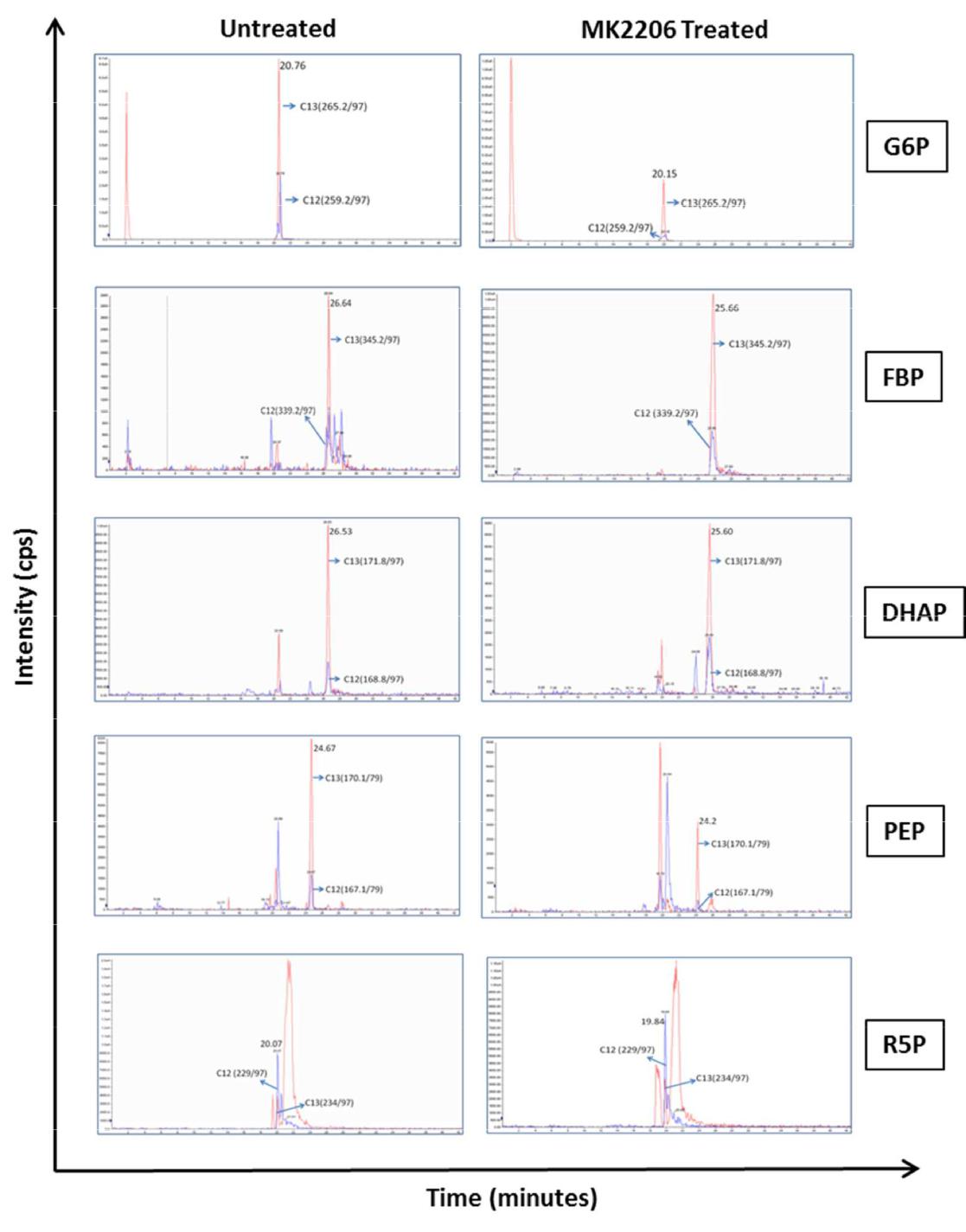


Figure S-9. Chromatograms are corresponding to ion spectra of palmitic acid (as standard) and free fatty acids. The peaks were obtained by direct infusion method in MK-2206 treated and untreated samples together with presence and absence of labeled carbon at 0 hours and 7 hours.


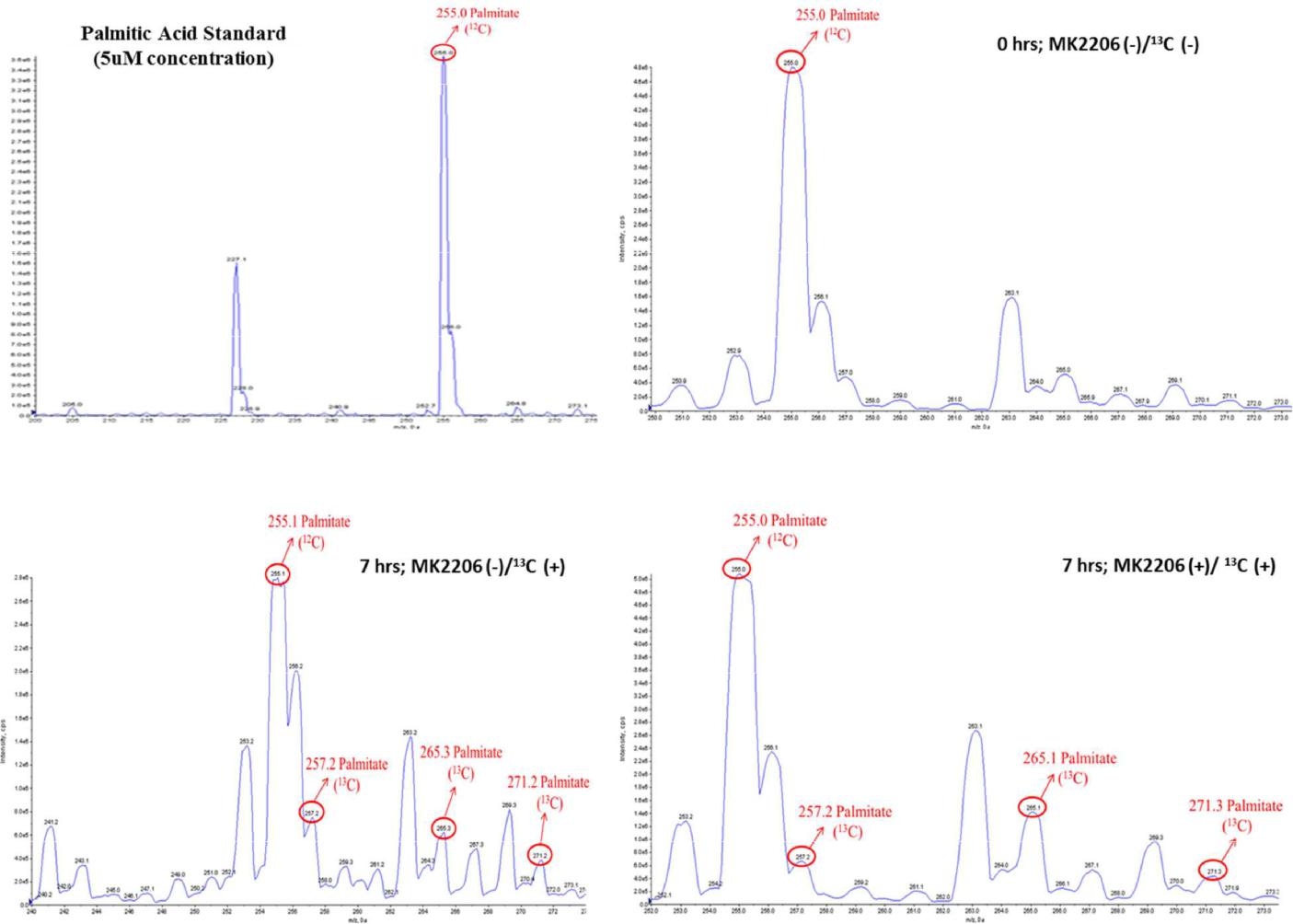


Figure S-10. Schematic diagram of the CCM pathways depicting uptake of glucose by the cells and its subsequent utilization across different metabolites. Proteins showing perturbed association with Akt1 inhibition and their corresponding reaction steps are highlighted in red colour in the figure.


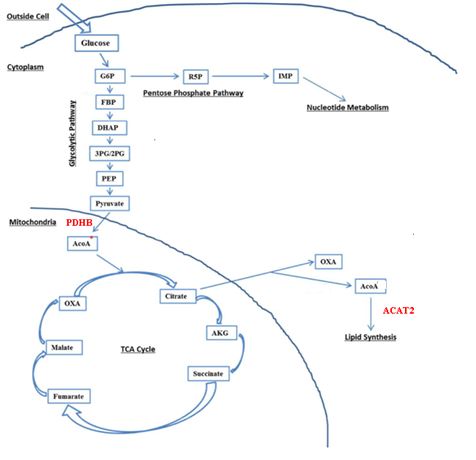

Supplement: Supplementary file 2 — Additional file 2: Figure S1. Detailed workflow to identify interacting partners of AKT1 followed by their subsequent analysis. Figure S2. Filters employed to generate the final list of AKT1 Interactors. Figure S3. KEGG pathway enrichment for AKT1 binding partners using WebGestalt. Figure S4. Validating the effect of AKT1 inhibitor (MK-2206) on AKT1 activity by western blotting. Figure S5. Specimen depiction of relative incorporation of labeled carbon in G6P and FBP metabolites in MK-2206 treated and untreated samples. Figure S6. Possible labeling pattern for key metabolite upon feeding cells with 13C6 Glucose. Figure S7. Specimen chromatograms depicting estimated false discovery rates in Lys C and Trypsin digested MK-2206 treated (Lys8Arg10) and untreated (Lys0Arg0) samples. Figure S8. Specimen chromatograms showing metabolite peaks in the untreated and MK-2206 treated samples for five different metabolites at one of the targeted time points. Figure S9. Chromatograms are corresponding to ion spectra of palmitic acid (as standard) and free fatty acids. Figure S10. Schematic diagram of the CCM pathways depicting uptake of glucose by the cells and its subsequent utilization across different metabolites. Proteins showing perturbed association with AKT1 inhibition and their corresponding reaction steps are highlighted in red colour in figure. [file 13104_2018_3364_MOESM2_ESM.docx]
